# Biosecurity Risk Factors and Predictive Index for Hepatitis E Virus Serological Status in Belgian Pig Farms: Conventional and Free-Range Systems

**DOI:** 10.3390/v17030432

**Published:** 2025-03-18

**Authors:** Constance Wielick, Louisa Ludwig-Begall, Stefaan Ribbens, Étienne Thiry, Christel Faes, Claude Saegerman

**Affiliations:** 1 Research Unit in Epidemiology and Risk Analysis Applied to Veterinary Sciences (UREAR ULiège), FARAH Research Centre, Department of Infectious and Parasitic Diseases, Faculty of Veterinary Medicine, University of Liège, 4000 Liège, Belgium; cwielick@uliege.be; 2 FARAH Research Centre, Veterinary Virology and Animal Viral Diseases, Department of Infectious and Parasitic Diseases, Faculty of Veterinary Medicine, University of Liège, 4000 Liège, Belgium; louisa.ludwig-begall@evidera.com (L.L.-B.); etienne.thiry@uliege.be (É.T.); 3 Animal Health Service Flanders (DGZ Vlaanderen), 8820 Torhout, Belgium; stefaan.ribbens@dgz.be; 4 Center for Statistics, Data Science Institute, Hasselt University, 3500 Hasselt, Belgium; christel.faes@uhasselt.be

**Keywords:** Hepatitis E, HEV, pig, swine, farm, seroprevalence, biosecurity, risk factors, protective factors, Belgium

## Abstract

Hepatitis E viruses (HEV) cause hepatitis E in humans. In industrialized countries, sporadic HEV infections, typically caused by HEV genotypes 3 or 4, can become chronic and progress to liver cirrhosis in immunocompromised individuals. Pigs are a significant animal reservoir, implicating raw or undercooked pork products as potential sources of human infection. To better understand HEV dissemination in the Belgian pig population, potential risk factors were investigated by linking farm-level HEV serological status to biosecurity questionnaire data. Farrow-to-finish herd type, free-range systems, and poor boot hygiene were significantly associated with higher within-herd prevalences. This enabled an initial risk profiling of various farming types and the development of predictions for all Belgian pig farms. When combined with the census of the Belgian wild boar population, the predicted HEV status of all professional Belgian pig farms (based on these associations) does not suggest that the proximity of wild boars is a main source of HEV in free-ranging herds. Identifying risk factors for increased circulation of HEV between and within pig farms is critical to controlling its spread and reducing human infection.

## 1. Introduction

Hepatitis E viruses (HEVs) are small, non-enveloped or quasi-enveloped, positive-sense, single-stranded RNA viruses within the Hepeviridae family [1] that cause hepatitis E disease in humans. According to the World Health Organization (WHO), HEV is responsible for approximately 20 million infections annually, 3.3 million of which are symptomatic [2]. Hepatitis E virus genotypes 1, 2, 3, 4, and 7 are known to infect humans [3,4,5]. Genotypes 1 and 2 are endemic in developing countries and are known to cause severe outbreaks of acute and fulminant hepatitis E, primarily through contaminated water sources (in Central and South Asia and large parts of Africa) [6,7,8]. In contrast, sporadic HEV infections in industrialized countries (such as Europe and North and South America) are typically caused by HEV 3 or 4 [9]. These infections are frequently asymptomatic and self-limiting; however, in immunocompromised individuals, a chronic form of the disease can lead to liver cirrhosis and associated neurological symptoms [8,10,11]. Consequently, HEV is increasingly recognized as a significant public health issue [10].

In Belgium, the last estimated seroprevalence of human HEV was 4.1% (95% CI: 3.1–5.1) in 2006 and 5.8% (95% CI: 4.8–6.9) in 2014 [12]. Pigs share HEV genotypes 3 and 4 with humans and are considered a major animal reservoir of the virus [13,14]. Although the exact routes of interspecies transmission are yet to be fully elucidated, growing evidence implicates the oral route, particularly through the consumption of raw or undercooked pig or game meat, in HEV infections [15]. Similarly to typically asymptomatic human HEV infections, HEV 3 and 4 are often silently spread amongst pig and wild boar populations [15,16,17,18,19]. Globally, pigs have been found to be highly HEV seroprevalent [16,17,18,19,20]. Importantly, animals have been found to carry HEV particles at slaughter, rendering raw or undercooked pig products as potential sources for human infection [21]. A first crucial step in monitoring the virus is identifying HEV-infected pig farms and potential risk factors for herd HEV seropositivity.

Through a large-scale, randomized, cross-sectional serological survey, we recently estimated HEV seroprevalence in Belgian pigs at 31% at the individual level and between 50% and 62% at the herd level [22]. These findings provide an overview of the current spread of HEV in Belgian pig herds. Using available demographic data from the sampled herds, an initial risk assessment of Belgian pig farms indicated widespread HEV circulation within the pig population. Factors such as herd size and herd type were identified as affecting a herd’s HEV serological status [22].

**Figure A1:**
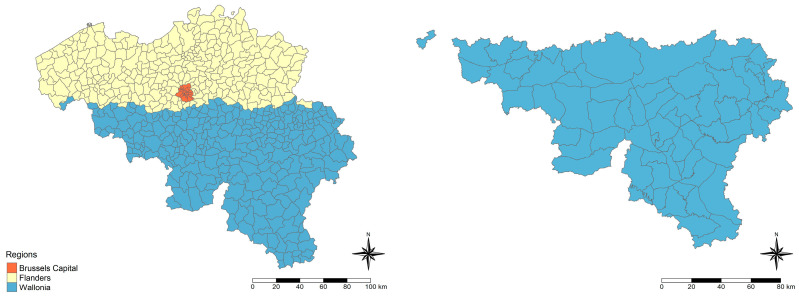
Belgian municipalities by region and hunting councils in 2021. The polygons on the map to the left represent the different Belgian municipalities in 2024, while the polygons on the right map depict the hunting councils in Wallonia in 2021.

Belgium is divided into three regions: the Brussels Capital Region, Flanders in the north, and Wallonia in the south, with the latter two regions each consisting of five provinces (Figure A1). Most professional pig production (active herds with at least 100 pigs) is concentrated in Flanders, accounting for 90.79% of herds [22]. The Belgian Federal Agency for the Safety of the Food Chain (Agence Fédérale pour la Sécurité de la Chaîne Alimentaire/Federaal Agentschap voor de Veiligheid van de Voedselketen (AFSCA/FAVV)) has assigned the responsibility for herd and animal identification and health to Animal Health Care Flanders (Dierengezondheidszorg Vlaanderen (DGZ)) and Animal Health Care Wallonia (Association Régionale de Santé et d’Identification Animales (ARSIA)) in their respective regions. The Belgian Identification and Registration System (SANITEL) serves as a centralized database for the computerized management of herd and animal identification, registration, and monitoring. Recently, the AFSCA/FAVV decreed that an official farm veterinarian must complete a biosecurity questionnaire for each pig herd via FarmFit, an official online application provided by the DGZ and ARSIA (Circular PCCB/S2/1690164, 31/05/2021, [23]).

Here, to enhance the current understanding of HEV dissemination in the Belgian pig population, we investigated potential risk factors by associating the HEV statuses (seropositive or seronegative) of farms with data obtained from the biosecurity questionnaire as exploratory variables. This enabled us to perform an initial risk profiling of various farming types and develop predictions for all Belgian pig farms. Based on these predictions, we created geographical visualizations.

Operating systems of free-range pig farms in which pigs are allowed outside during rearing are a recent development in pig production [24]. These systems are believed to affect HEV dynamics in herds [25,26]. Differences from more traditional professional systems include farming intensity and density (pigs per square meter), environmental factors (e.g., fecal distribution and cleaning practices), and the potential for contact with wild boar populations [27]. Our previous investigation into whether free-range systems increase the risk of HEV herd seropositivity did not find a significant impact of such systems on HEV herd serological status [22], as there were not enough farms to draw firm conclusions. In this study, the availability of data for all Belgian pig farms, along with the predictions of their HEV serological status, were leveraged to evaluate the proximity of Belgian pig farms to the HEV wild boar reservoir and its potential burden on the pig population.

## 2. Materials and Methods

### 2.1. Serological Data

Raw serological data were retrieved from a randomized, robust, large-scale, cross-sectional, serological survey of HEV infection previously performed by our group and investigating the dissemination of HEV in Belgian pig farms between 2020 and 2021 [22]. Briefly, the population of Belgian pigs, specifically those housed in herds comprising at least 100 animals, was randomly stratified according to geographical localization (ten Belgian provinces) and herd type (mixed farrow-to-finish herd, closed farrow-to-finish herd, slaughter pig herd, and “other” including quarantine farms, breeding stock farms). In total, 266 herds were randomly selected. The samples were collected by the farms’ veterinarians as part of the Belgian national Aujeszky’s disease control plan. The sampling method was established by the ministerial decree of 23 July 2013 (Numac code: 201301834) [28]. Six sera from adult sows or twelve sera from the oldest or heaviest fattening pigs of each herd were tested using a double-antigen sandwich enzyme-linked immunosorbent assay (ELISA) (HEV ELISA kit 4.0 V, MP Biomedicals, Eschwege, Germany) to detect total anti-HEV antibodies. A herd was considered positive as soon as one sampled serum tested positive.

### 2.2. Biosecurity Questionnaire

To evaluate the relationship between the serological data and various exploratory variables, we took advantage of the existence of a recent state-mandated online questionnaire (FarmFit; DGZ/ARSIA) by pairing it with the serological survey on HEV herd-infection. Based on a scientific risk-based scoring system to evaluate on-farm biosecurity (Biocheck.UGent platform; developed by the University of Gent [29]), the DGZ/ARSIA FarmFit tool records the general biosecurity measures applied on a given farm via multiple-choice questions. It calculates biosecurity scores based on the answers (internal, external, and general biosecurity scores) [30]. These scores are determined by assigning a relative importance to each possible form of disease transmission, based on literature data and general knowledge about the probabilities of disease introduction and spread. The final score is also determined by the frequency of occurrence of the specific transmission routes. This risk analysis of pathogen introduction and spread is generic and is not specifically designed for a particular pathogen.

Authorization to anonymously transfer the biosecurity data from the selected herds in this study (reported between June and November 2021) was obtained by DGZ from the AFSCA for analysis. Of the 266 initially selected herds, the data for four were missing (their owners or the responsible veterinarians had not replied to the questionnaire), and five herd proprietors had stopped their occupation at the time of retrieval. A total of 257 herds were therefore included in the analysis. The biosecurity questionnaire is based on the Biocheck and is available online [29]. It comprises a total of 142 questions, which were classified into five different categories of biosecurity measures, as defined by Renault et al. [31]: bio-exclusion (measures preventing the introduction of an infectious agent into the farm), bio-compartmentalization (measures preventing the spread and dissemination of a pathogen among the farm’s animals), bio-containment (measures preventing the inter-farm transmission of a pathogen), bio-prevention (measures preventing the transmission of zoonotic pathogens to humans), and bio-preservation (measures preventing contamination of plants and/or the environment). Some biosecurity measures can be classified into multiple compartments. Since the only data available for evaluation of these measures in the context of the present study were the herd- and within-herd HEV prevalences, only questions pertaining to bio-exclusion and bio-compartmentalization measures were retained for analysis.

Based on the analysis of the sampled herds and to predict the current HEV serological statuses of pig farms at a national Belgian scale, the answers to questions of interest for all active pig farms in Belgium in April 2024 were retrieved (anonymized retrieval) together with their Lambert conformal conic projection.

### 2.3. Statistical Analysis

Only discrete exploratory variables were retained, and variables where answers/categories comprised less than 15 herds were considered not interpretable and were excluded from the analysis. The bio-exclusion and bio-compartmentalization measures were assessed using the HEV-within-herd prevalences previously estimated [22] using a beta-binomial model considering the ELISA assay’s sensitivity (Se) and specificity (Sp), within-herd correlation, the size of the sampled herds, and the age of the sampled pigs, as described by Faes et al. [32]. Herds for which two different age categories of pigs were tested were assigned two estimated within-herd prevalences and were thus represented twice in the analysis. The final analysis was performed on 267 herds in total. The estimated within-herd prevalences were categorized into two modalities: lower than and equal to or higher than 50% HEV-within-herd prevalence. A kernel density estimation was performed using STATA/SE Acad. 14.2 (Stata Corp., College Station, TX, USA) to determine this 50% cut-off (CO). The relationship between HEV serological status (<50% versus ≥50% within-herd prevalence) and different exploratory variables was assessed using the odd ratios (OR) that were estimated using logistic regression, followed by a forward automated selection of variables based on the Bayesian information criterion (BIC) using the “step” function using R version 4.1.2 software.

Based on both the bio-exclusion and bio-compartmentalization models, a predictive index (PI) was calculated for each herd using the following formula:

(1) $$\mathrm{PI}=\sum_{i=1}^{n}(F_{R;i}\times {OR}_{R;i})$$

where F_R_ is the risk factor (0 or 1 for absent or present), and OR_R_ is the odds ratio of the risk factor [33]. To evaluate the accuracy of this method to discriminate between low- and high-HEV-prevalence herds, a receiver operating characteristic (ROC) curve was built and its area under the curve calculated using STATA/SE Acad. 14.2 (Stata Corp., College Station, TX, USA). For each CO point of the PI, the Youden’s index (*Se* + *Sp* − 1), which determines the CO point optimizing the test Se and Sp [34], was calculated. The maximum Youden’s index, *max* (*Se* + *Sp* − 1), was used to determine the optimal CO value for the PI [35,36]. Finally, the PI and its optimal CO were applied to predict the HEV serological statuses of all Belgian pig farms based on the answers to the questions of interest raised in the questionnaire.

### 2.4. Spatial Visualization

The professional pig farms (active herds with at least 100 pigs in 2021) and their predicted HEV within-herd prevalences were spatially represented and analyzed using R 4.3.3 software (sf, tmap, tmaptools, tidyverse, geodata, and janitor packages). To calculate the ratios between the number of farms, if the denominator was zero, the ratio was assigned the value of the numerator. The spatial data, forest areas, and number of wild boar hunting bags were provided by the Public Service of Wallonia (SPW) and by the Instituut voor Natuur- en Bosonderzoek (INBO). In Flanders (INBO), the data are available for each municipality. In Wallonia (SPW), the data are collected at the level of the hunting council. These hunting councils, 68 in total, are non-profit associations established by hunters to coordinate game management across various territories within a given area (Figure A1; right map).

## 3. Results

### 3.1. Bio-Exclusion: Piglet Purchase Protects Against Increased HEV Herd Seroprevalence

**Figure 1:**
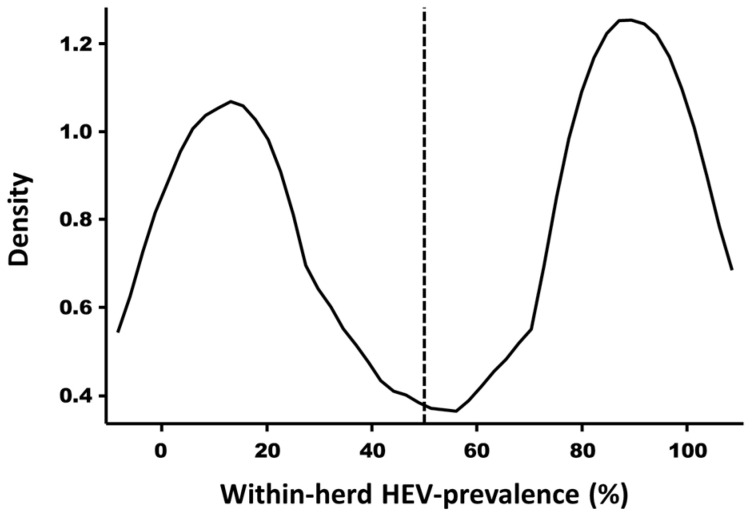
Kernel density estimate (using the Epanechnikov function) of the within-herd hepatitis E virus (HEV) prevalences of sampled herds. The red vertical bar represents the chosen cut-off (CO) of 50%; bandwidth = 0.1105.

Based on the estimated within-herd HEV prevalences, the kernel density estimate showed a clear separation of the sampled herds into two populations (Figure 1): those with a within-herd prevalence lower than 50% and those with a within-herd prevalence equal to or higher than 50%. We therefore decided to use this CO to sort the herds into two categories of HEV serological status.

No association was observed between the different biosecurity scores (internal, external, and general biosecurity scores) calculated via the Biocheck method [30] and the HEV serological statuses.

Seventy-seven questions addressing measures preventing the introduction of an infectious agent into the farm were classified as part of the bio-exclusion compartment. Of these, 34 questions were included in the analysis (Table A1). The best final selected model (according to the automatic model selection using the BIC) was a univariate model comprising the purchase of piglets as a protective factor for HEV herd serological prevalence (Table 1). Herds that purchase piglets rather than having them born on the farm were significantly less at risk of having high HEV within-herd prevalences.

### 3.2. Bio-Compartmentalization: Free-Range Systems and Low Boot Hygiene Are Risk Factors for Increased HEV Herd Seroprevalence

Sixty-five questions dealing with measures preventing the inter-animal spread and dissemination of a pathogen on a given farm were classified as part of the bio-compartmentalization category. Of these, 32 questions were included in the analysis (Table A2).

The best final selected model (according to the automatic model selection using the BIC) was a multivariate model comprising the availability of a free-range system and the absence of either a footbath or a boot-washer or the changing of boots between compartments as risk factors for increased HEV herd serological prevalence (Table 2). Herds that allow pigs access to a free-range system and do not have a footbath or boot washer or do not change boots between compartments have a higher HEV within-herd prevalence.

### 3.3. Prediction of the HEV Serological Status of Belgian Pig Farms and Spatial Visualization

**Figure 2:**
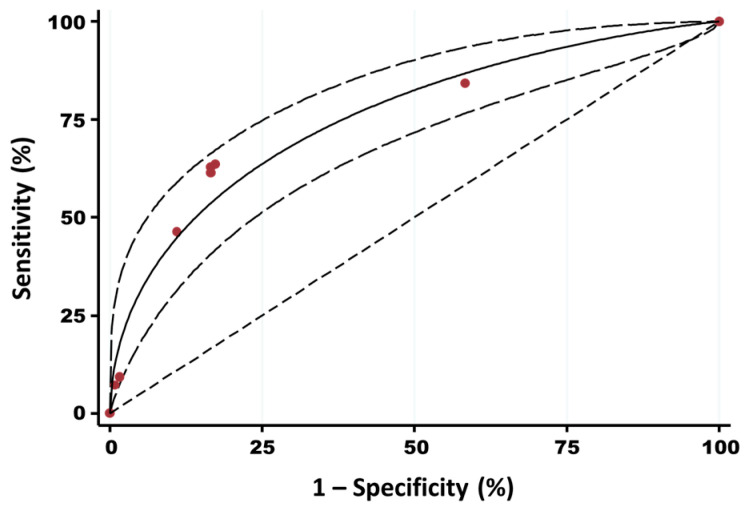
Receiving operating characteristic (ROC) curve of the index of the sampled herds versus their estimated within-herd HEV seroprevalence (< or ≥50%). Area under curve = 0.76; standard error (area) = 0.0307; red dots: CO points of the index; solid line: fitted ROC curve (fitting binormal model); dashed lines: 95% confidence interval of the fitted ROC curve; dotted line: reference line representing the area under the curve of 0.5.

Based on both the bio-exclusion and bio-compartmentalization models, a PI was calculated for each herd of the sample. The ROC curve built based on these results has an area under the curve of 0.76, indicating that the PI has an acceptable accuracy to predict the HEV within-herd seroprevalence (<50% vs. ≥50% HEV seroprevalence), as defined by Swets [37] (Figure 2). The Youden’s index shows that 2.55 is the best CO point of the PI to discriminate between <50% and ≥50% of HEV within-herd seroprevalence.

**Figure 3:**
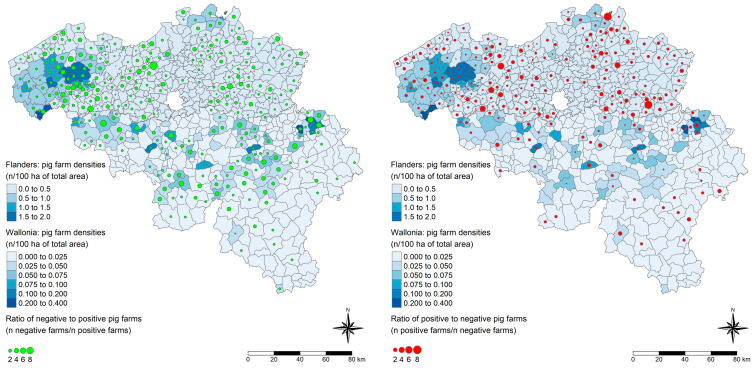
Distribution of pig farm densities and ratios of predicted HEV statuses of pig farms in Belgium, 2021. The left panel illustrates the ratios of negative to positive pig farms (number (n) negative farms/n positive farms). The right panel illustrates the ratios of positive to negative pig farms (n positive farms/n negative farms). Negative statuses are defined as farms with less than 50% estimated within-herd prevalence, while positive farms are defined as farms with 50% or higher estimated within-herd prevalence. Pig farm densities are expressed as the number (n) of farms per 100 hectares (ha) of the municipality’s total area. A different legend scale is used for Wallonia to enhance contrasts. The polygons on the maps represent the Belgian municipalities in 2021.

Finally, the PI and its optimal CO were applied to predict the HEV serological statuses of all Belgian pig farms based on all answers to the questionnaire’s questions of interest. The distribution of pig farm densities (number of farms per 100 ha of total area) and the ratios of HEV statuses (less than 50% estimated within-herd prevalence and equal or higher than 50% estimated within-herd prevalence) of pig farms were compared (Figure 3). Contrary to expectations, a higher number of farms with low within-herd prevalences were found in areas with high farm density; this was particularly noticeable in western Flanders.

**Figure 4:**
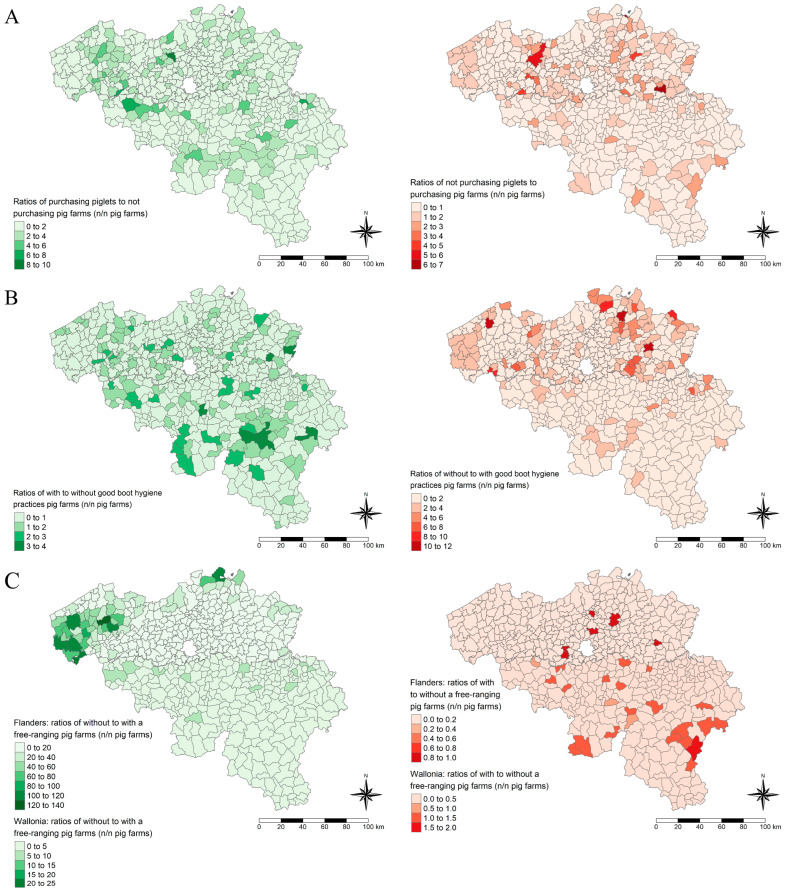
Distribution of pig farm ratios based on various variables: piglet purchase, availability of a free-range system, and the absence of footbaths, boot-washers, or changing boots between compartments. Panel (**A**) shows the distribution of the ratio of farms that purchase piglets to those that do not (green) and the ratio of farms that do not purchase piglets to those that do (red). Panel (**B**) shows the distribution of the ratio of farms with good boot hygiene practices to those without (green) and the ratio of farms without good boot hygiene practices to those with (red). Panel (**C**) displays the distribution of the ratio of farms without a free-range system to those with (green) and the ratio of farms with a free-range system to those without (red), using a different legend scale for Wallonia to enhance contrasts. Ratios are expressed as the number of farms with a risk or protective factor to the number of farms with a protective or risk factor (n farms/n farms). The polygons on the maps represent the Belgian municipalities and are colored green if the numerator of the ratio represents farms with a protective factor for increased predicted HEV within-herd seroprevalence and red if the numerator of the ratio represents farms with a risk factor for increased predicted HEV within-herd seroprevalence.

The distribution of all Belgian pig farms is represented based on three variables included in the final models (Figure 4): piglet purchase, the availability of a free-range system, and the absence of footbaths, boot-washers, or the changing of boots between compartments. Notably, purchasing young piglets was strongly associated with the adoption of effective boot hygiene practices (*p*-value < 0.05, OR = 2.01, 95% CI = 1.76–2.28).

**Figure 5:**
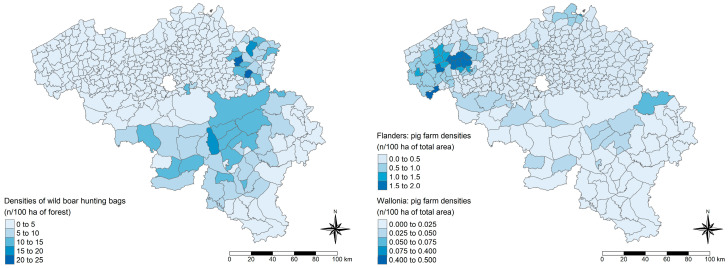
Distribution of wild boar and pig farm densities in Belgium in 2021. The left panel shows the distribution of wild boar hunting-bag densities (number (n) of hunting bags per 100 ha of forest area) in Belgium in 2021. The right panel depicts the distribution of pig farm densities (number (n) of farms per 100 ha of total area) in Belgium during the same year, using a different legend scale for Wallonia to enhance contrasts. The polygons on the maps represent the Belgian municipalities in Flanders and the hunting councils in Wallonia.

To evaluate the proximity of the two main HEV reservoirs (domestic pigs and wild boars), data from the census of the wild boar population in Belgium were combined with pig farm densities for the same year (2021). Wild boar hunting-bag densities (number of hunting bags per 100 ha of forest area) and pig farm densities (number of pig farms per 100 ha of total area) in Belgium in 2021 were compared (Figure 5), showing that the densities of wild boars (left map) and pig farms (right map) do not geographically coincide (correlation = −0.20, *p*-value < 0.05).

**Figure 6:**
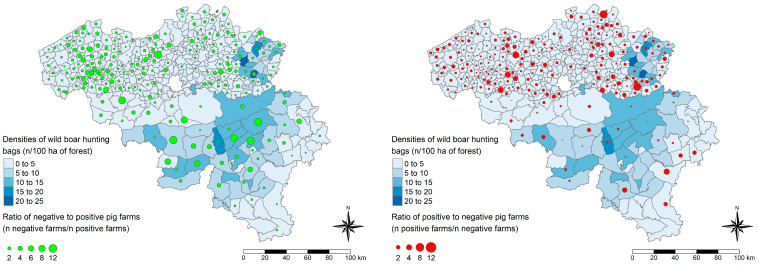
Distribution of wild boar densities and ratios of predicted HEV statuses of pig farms in Belgium, 2021. Left panel illustrates the ratios of negative to positive pig farms (n negative farms/n positive farms). Right panel illustrates the ratios of positive to negative pig farms (n positive farms/n negative farms). Negative statuses are defined as farms with less than 50% estimated within-herd prevalence, while positive farms are defined as farms with 50% or higher estimated within-herd prevalence. The polygons on the maps represent the Belgian municipalities in Flanders and the hunting councils in Wallonia. Wild boar hunting-bag densities are expressed as the number (n) of hunting bags per 100 hectares (ha) of forest area per municipality (Flanders) or hunting council (Wallonia).

In areas with high wild boar densities, a comparison of the wild boar densities (number of wild boar hunting bags per 100 ha of forest area) and the HEV status ratios of pig farms (less than 50% estimated within-herd prevalence and equal or higher than 50% estimated within-prevalence) reveals that farms with low within-herd prevalence are more common than those with high prevalence (correlation = 0.13, *p*-value < 0.05; Figure 6). When comparing the proximity between farms and wild boar populations, expressed as the ratio of the number of wild boars to the number of pig herds, with the ratios of HEV statuses in each area, no statistically significant impact of this proximity on the predicted HEV statuses of pig herds was observed.

**Figure 7:**
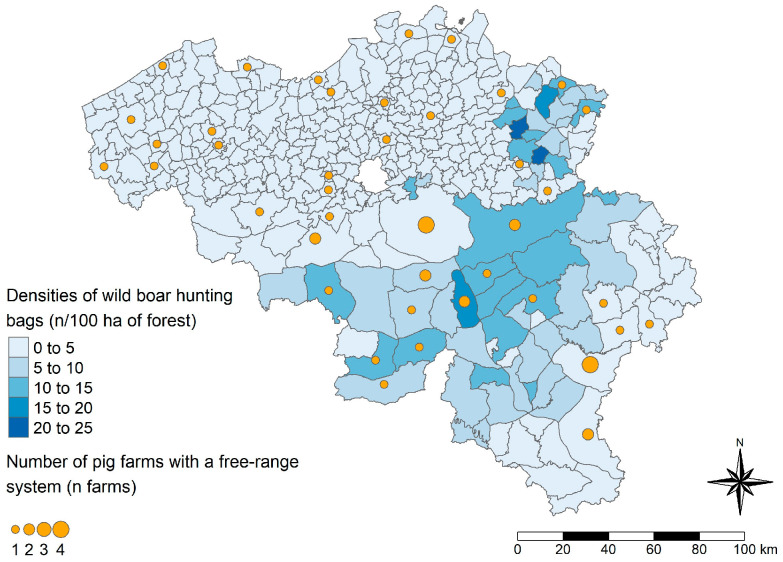
Distribution of wild boar densities and free-ranging pig farms in Belgium, 2021. Wild boar hunting-bag densities are expressed as the number (n) of hunting bags per 100 hectares (ha) of forest area per municipality (Flanders) or hunting council (Wallonia). The polygons on the maps represent the Belgian municipalities in Flanders and the hunting councils in Wallonia.

Farms operating a free-range system are mostly located in areas where fewer wild boar hunting bags have been reported and are distributed all over Belgium (Figure 7).

## 4. Discussion

In this study, we investigated various exploratory variables to identify factors that favor the spread and dissemination of HEV within the Belgian pig population, using previously identified within-herd HEV serological prevalences [22]. These variables relate to biosecurity measures: either bio-exclusion (preventing the introduction of an infectious agent into the farm) or bio-compartmentalizing (preventing the spread and dissemination of a pathogen among the farm’s animals). Our results show that the farrow-to-finish herd type (bio-exclusion) and the presence of a free-range system and poor boot hygiene (bio-compartmentalization) are significantly associated with higher within-herd prevalences. When combined with the census of the Belgian wild boar population, the predicted HEV status of all professional Belgian pig farms (based on these associations) does not suggest the proximity of wild boars is a potential source of HEV in free-ranging herds.

To evaluate bio-exclusion, i.e., measures preventing the introduction of an infectious agent into the farm, the herd’s serological status (positive versus negative) is typically the most appropriate parameter to use. However, to anticipate a possible lack of accuracy of the ELISA assay used (Ss and Sp issues) as well as the limited number of animals tested per herd (6 or 12 animals per herd), we instead decided to use estimated within-herd prevalences and their 50% CO (based on the kernel density estimation; Figure 1).

After automatic model selection, the analysis of 34 variables related to bio-exclusion measures (Table A1) identified the purchase of piglets as the only significant protective factor for increased HEV herd serological prevalence. This outcome reinforces previous observations based solely on available demographic data from the SANITEL database and apparent herd seroprevalences [22].

Herds that purchase piglets are typically slaughter pig herds. Moreover, the data from the biosecurity questionnaire are more up-to-date and therefore more accurate, as they stem from the recently mandated biosecurity questionnaire administered by farmers’ official veterinarians [23]. In contrast, the SANITEL database is not updated and does not reflect changes occurring on the farm since registration, as farmers do not report changes in management. Consequently, the findings derived from the demographic data and apparent herd-seroprevalences, as well as those based on the estimated within-herd prevalences and the biosecurity questionnaire, are entirely consistent.

While this finding may seem unexpected (the arrival of new pigs is commonly recognized as a way for infectious pathogens to enter pig farms [38]), we and multiple other authors have previously shown that maternal antibodies are transmitted from sows to their offspring [22,39,40,41]. We hypothesize that sows constitute a reservoir for HEV on farms, leading to a high transmission of maternal antibodies to piglets at birth. Piglets transferred to finishing farms are likely still protected against HEV infection upon arrival at a new location to start their finishing. As such, they arrive on a site without any HEV reservoir (i.e., sows), resulting in a less contaminated (and less often re-contaminated) environment. In slaughter pig farms, the widespread presence of protective maternal antibodies may thus protect piglets until after purchase or transfer to a new housing location (potentially free of HEV) for finishing. This could potentially slow down or even prevent HEV infection. In contrast, in farrow-to-finish pig herds, although high maternal antibody levels are present, piglets remain on the farm until slaughter, and the continuous presence of older pigs (potential HEV reservoirs) may lead to constant contamination and recontamination.

The sampling protocol used constitutes a potential bias. The serum samples analyzed were selected from those collected within the framework of the Belgian national Aujeszky’s disease control plan, as defined by the ministerial decree of 23 July 2013 (Numac code: 2013018341 [28]). As farrow-to-finish farms house older pigs (i.e., sows), the probability of finding seropositive pigs is increased in these farms [22]. To mitigate the effect of the sampled pigs’ age and adopt a more conservative approach, estimated within-herd seroprevalences were used. These estimates were based on a beta-binomial model that corrected for the ELISA assay’s sensibility and specificity, within-herd correlation, the size of the sampled herds, and the age of the sampled pigs, using a 50% CO for herd categorization (Figure 1). Nevertheless, further investigations encompassing sampling all pigs at the same age (e.g., slaughter age) are needed to suppress the influence of sampling age on the HEV statuses of different herd types.

To assess the relationship between HEV serological status (<50% versus ≥50% within-herd prevalence) and different bio-compartmentalizing measures preventing the inter-animal spread and dissemination of a pathogen on a given farm, 32 exploratory variables were analyzed (Table A2). The final selected model identified the absence of either a footbath, a boot-washer, or the changing of boots between compartments and the availability of a free-range system as risk factors for increased HEV herd seroprevalence.

Adopting effective boot hygiene practices was significantly associated with purchasing young piglets. Conversely, farms that do not purchase piglets, such as farrow-to-finishing farms, seem to implement less rigorous boot hygiene. The exact reason for this observation is unclear, as the maternity stage is likely the riskiest period in the piglets’ lives and should be the most targeted stage for good hygiene practices. One possible explanation could be that many finishing farms rear only one batch at a time using an all-in, all-out practice. They might consider that they have only one compartment, and the boots are specific to that compartment.

The implementation of measures to ensure boot hygiene was identified as a protective factor against increased within-herd seroprevalence. This suggests that higher levels of boot hygiene correspond to lower levels of HEV circulation within the farm. Walachowski’s study demonstrated that poor boot hygiene had a significant positive effect on the percentage of HEV-positive livers at slaughter, hypothesizing an increased spread of HEV among batches of pigs [42]. HEV can be present on boots [43], allowing for transmission between pens. Whether this specific measure is responsible for the higher circulation of HEV in farrow-to-finishing farms or if it merely reflects the general level of internal biosecurity on the farm remains to be determined.

The second bio-compartmentalization measure significantly related to HEV serological status (<50% versus ≥50% within-herd prevalence) was the availability of a free-range system. Free-range systems have been suggested to influence HEV herd dynamics through possible contacts with wild animal reservoirs [25,26]. Therefore, we investigated the proximity of such farms to the wild boar population in Belgium at the time of the study. Generally, Belgian pig farms were located in Flanders, while areas most densely populated with wild boars were mainly in Wallonia (Figure 5). In areas with high wild boar densities, there were more farms with low within-herd HEV prevalences than farms with high within-herd HEV prevalences (Figure 6). Farms operating a free-range system were mostly located in areas where fewer wild boar hunting bags were reported in 2021, and these farms were distributed throughout Belgium. Based on these geographical comparisons, we were not able to show a statistically significant influence of the proximity between the wild boar and domestic pig populations on predicted herd HEV statuses. However, we observed that the risk of contact between the two reservoirs was limited. Since the African swine fever crisis in 2018–2019 [44], this risk limitation might have been intensified. Wild boar populations and their movements have been managed both in Belgium and in neighboring France [45,46]. Further investigation, e.g., via genomic analyses of samples from adjacent populations of pigs and wild boars, is needed to confirm the role of wild boars in the spread of HEV among domestic pigs. Given that these two populations have opposing geographic distributions in Belgium, it would be more appropriate to study the role of wild boars in the spread of HEV in other regions where the two populations are in close proximity.

Potential contacts with wild animal reservoirs are not the only possible explanation for the relationship between HEV statuses and free-range systems. Differences from more traditional professional systems also include environmental factors such as fecal distribution and cleaning. In free-range farms, pigs are typically kept on a mix of straw and an outdoor parcours (usually on cement) or in fields. In the latter case, the fields cannot be cleaned and disinfected, and feces decompose directly on the ground. The high resistance and stability of HEV to various inactivation methods have been demonstrated [47,48,49]. Given the high persistence of HEV, it is easy to envisage the accumulation of the virus in the environment under such conditions. These setups likely create ideal conditions for facilitating contact with contaminated feces as opposed to those with slatted floors. Andraud et al. showed that the levels of environmental contamination resulting from fecal shedding had an impact on the spread of HEV inside the pen [50]. This was further supported by Walachowski et al., who showed an increased spread of HEV when pigs were closer to the manure pit, suggesting more frequent contact with contaminated feces [42]. Oro–fecal transmission of HEV in pigs requires repeated exposures to high doses of the virus, according to Kasorndorkbua et al. [51]. Settings that facilitate environmental accumulation of HEV particles are likely to promote continuous circulation of HEV between batches.

Several studies have investigated the influence of biosecurity measures on HEV spread. Environmental contamination has been identified as a significant source of infection and potentially re-infection of pigs [25,26,42,50,52,53]. We were not able to show a correlation between internal or external biosecurity scores calculated from the original questionnaire and estimated within-herd prevalences. This may be due to the fact that scoring of different questionnaire sections might not be adequately tailored to HEV. (We were unable to verify this since the calculation of the scores is confidential.) One of the limitations of this cross-sectional study is that it does not allow for the establishment of causality. While associations between exposure and outcomes can be identified, temporal relationships cannot be determined. Future cohort studies or intervention studies would be required to test the causal hypothesis and confirm the findings of this study. The use of the state-mandated, country-wide questionnaire nevertheless represents a strength of this exploratory study.

## 5. Conclusions

Identifying risk factors for increased circulation of HEV between and within pig farms is a crucial step in controlling HEV spread and reducing human infection. While biosecurity measures addressing environmental contamination play a role in HEV transmission, the exact mechanisms are likely complex and require further investigation. The findings of this study in Belgium are relevant to other European countries given the similarities in pig production systems and swine husbandry practices across Western Europe. Our results establish a baseline for future studies aiming to describe the dynamics of HEV infection in pigs and the development of measures to mitigate human infection.

## Figures and Tables

**Table 1:** Effect of different bio-exclusive measures on the within-herd HEV seroprevalence of 267 Belgian farms.

Variable	Category	N	Univariate Model	
			OR (95% CI)	*p*-Value
Piglets’ purchase	Yes	160	-	-
	No	107	8.03 (4.51–14.34)	<0.01 ***

Effect of different bio-exclusive measures on the within-herd HEV seroprevalence of 267 farms. The odd ratios (OR) were determined by logit regression, followed by an automatic model selection using the Bayesian information criterion (BIC). Variables with a *p*-value < 0.05 were considered significantly related to herd HEV serological status. For *p*-value, *** refers to *p*-value < 0.01. Abbreviations: CI, confidence interval; N, number of Belgian pig farms investigated.

**Table 2:** Effect of different bio-compartmentalizing measures on the within-herd HEV seroprevalence of 267 Belgian farms.

Variable	Category	N	Univariate Model		Multivariate Model	
			OR (95% CI)	*p*-Value	OR (95% CI)	*p*-Value
Availability of a free-range system	Yes	19	1.41 (1.12–1.77)	0.004 ***	1.39 (1.10–1.74)	0.005 ***
	No	248	-	-	-	-
Presence of a footbath/boot-washer or changing of boots between compartments	Yes	109	-	-	-	-
	No	158	1.17 (1.04–1.32)	0.01 **	1.16 (1.03–1.31)	0.016 **

Effect of different bio-compartmentalizing measures on the within-herd HEV seroprevalence of 267 farms. The OR were determined by logit regression, followed by an automatic model selection using the BIC. Variables with a *p*-value < 0.05 were considered significantly related to herd HEV serological status. For *p*-value, ** refers to *p*-value < 0.05 and *** refers to *p*-value < 0.01.Abbreviations: CI, confidence interval; N, number of Belgian pig farms investigated.

## Data Availability

Data are contained within article.

## Abbreviations

The following abbreviations are used in this manuscript:

HEV	Hepatitis E virus
WHO	World Health Organization
AFSCA/FAVV	Agence Fédérale pour la Sécurité de la Chaîne Alimentaire/Federaal Agentschap voor de Veiligheid van de Voedselketen
DGZ	Dierengezondheidszorg Vlaanderen
ARSIA	Animal Health Care Wallonia (Association Régionale de Santé et d’Identification Animales
SANITEL	Belgian Identification and Registration System
ELISA	Enzyme-linked immunosorbent assay
Se	Sensitivity
Sp	Specificity
OR	Odds ratio
BIC	Bayesian information criterion
PI	Predictive index
ROC	Receiver operating characteristic
CO	Cut-off
SPW	Public Service of Wallonia
INBO	Instituut voor Natuur- en Bosonderzoek

## Appendix B

**Table A1:** List of exploratory variables included in the automatic model selection analysis for the bio-exclusion compartment.

Variable	Category
Type	Closed f-to-f herd
	Mixed f-to-f herd
	Slaughter pig herd
	Other
Size	101–200
	201–500
	501–1000
	1001–2000
	2001–4000
	>4000
Internal biosecurity score	
External biosecurity score	
General biosecurity score	
Do the pigs have access to free range?	Yes
	No
Besides pigs, are there any other livestock animals on your farm?	Yes
	No
Are there any cattle on the farm?	Yes
	No
Do you purchase breeding pigs (sows/gilts/boars)?	Yes
	No
Are piglets purchased?	Yes
	No
When purchasing semen, is there proof that the health status and health management of the originating AI center/company are equal to or superior to those of your own farm?	Yes
	No
	Not known
	No purchase of semen
Is the transport vehicle bringing pigs to the slaughterhouse or other farms empty upon arrival at the farm, and are the animals loaded from an isolated loading dock or directly from the barn/central corridor?	Central corridor and empty vehicle
	Central corridor and not empty vehicle
	Loading dock or no transport
Is the manure/slurry removed through the dirty area of the farm?	Yes
	No
Are there specific suction pipes on the farm for removing slurry from the pit (permanent slurry removal pipes on the farm)?	Yes
	No
Do the rendering company or the feed supplier handle carcass removal or silo filling without entering the farm buildings?	Yes
	No
Does the farmer use a feed supplier that meets specific hygiene requirements (e.g., Salmonella-free, heat treatment)?	Yes
	No or not known
Is a bacteriological analysis of the drinking water conducted annually at the source and the storage tank?	Yes
	No
Is a bacteriological analysis of the drinking water conducted annually at the main outlets (where the animals drink)?	Yes
	No
Are specific measures taken for the supply of equipment (e.g., cleaning and disinfection, quarantine period in a designated area)?	Yes
	No
Is a period of no contact with pigs (more than 12 h) required for all visitors before they are allowed to enter the barns?	Yes
	No
Is there a hygiene lock available, and is it always used when visitors access the livestock buildings?	Yes
	No
Are all livestock buildings accessible to visitors only through the hygiene lock?	Yes
	No
	No hygiene lock
Is vermin (rats, mice, etc.) considered a problem on the farm?	Yes
	No
Are the surroundings of the farm (nearby buildings) paved and kept clean (e.g., waste removal, control of weeds) to prevent infestations by rodents/wild animals?	Yes
	No
Do pets (dogs or cats) have access to the livestock buildings?	Yes
	No
Can birds enter the livestock buildings?	Yes
	No
Are there grilles placed at the air inlets?	Yes
	No
Is the farm located in an area with low or high pig density?	High
	Low
Are there any other pig farms within a 500 m radius of your farm?	Yes
	No
Is slurry from other farms spread on neighboring land directly adjacent to the livestock buildings (within a 500 m radius)?	Yes
	No
Are vehicles transporting animals from other farms frequently (at least once a day) passing on the road near the farm?	Yes
	No
Have wild boars been spotted in the vicinity of the farm (within a 10 km radius)?	Yes
	No
Are footbaths present at the entrance of the farm, and are they used?	Yes
	No
Are the buildings constructed to prevent wild boars from intruding or coming into contact with the pigs?	Yes
	No

List of exploratory variables included in the automatic model selection analysis for the bio-exclusion compartment. Bio-exclusive measures are measures preventing the introduction of an infectious agent into the farm. Abbreviation: f-to-f, farrow-to-finish.

**Table A2:** List of exploratory variables included in the automatic model selection analysis for the bio-compartmentalization compartment.

Variable	Category
Size	101–200
	201–500
	501–1000
	1001–2000
	2001–4000
	>4000
Internal biosecurity score	
External biosecurity score	
General biosecurity score	
Do the pigs have access to free range?	Yes
	No
Are there any cattle on the farm?	Yes
	No
Are the animals loaded from an isolated loading dock or directly from the barn/central corridor?	Central corridor or barn
	Loading dock
Is the manure/slurry removed through the dirty area of the farm?	Yes
	No
Are there specific suction pipes on the farm for removing slurry from the pit (permanent slurry removal pipes on the farm)?	Yes
	No
Is the carcass storage site secured to prevent access by dogs, cats, rodents, or wild animals?	Yes
	No
Does the farmer wear disposable gloves when handling carcasses or wash and disinfect their hands afterward?	Never
	Sometimes
	Always
Is it required to wash and disinfect hands before entering the livestock buildings?	Yes
	No
Is vermin (rats, mice, etc.) considered a problem on the farm?	Yes
	No
Are the surroundings of the farm (near the buildings) paved and kept clean (e.g., waste removal, weed control) to prevent infestations by rodents/wild animals?	Yes
	No
Do pets (dogs or cats) have access to the livestock buildings?	Yes
	No
Can birds enter the livestock buildings?	Yes
	No
Are there grilles placed at the air inlets?	Yes
	No
Is the health status of the farm (e.g., serology, reasons for seizure at the slaughterhouse, etc.) evaluated regularly (i.e., at least once a year)?	Yes
	No
Are sick animals always handled after healthy animals?	Yes
	No
Are specific clothing and footwear for each compartment available and changed between compartments?	Never
	Sometimes
	Always
Are hands washed and/or disinfected between livestock compartments?	Never
	Sometimes
	Always
Are footbaths/boot washers placed between different compartments, or are boots changed between compartments?	Yes
	No
On the farm, is all work conducted from the youngest pigs to the oldest?	Yes
	No
Is the equipment required for a particular group of animals arranged according to the work order to avoid using the same equipment across different groups?	Yes
	No
Is there a cleaning and disinfection protocol for equipment (e.g., brooms, shovels) after use, and is this protocol followed?	Yes
	No
Is the equipment clearly identified or recognizable (e.g., by color coding) as being reserved for a specific area or age group?	Yes
	No
Are syringes (for injections) available and used specifically for each age group?	Yes
	No
Are needles (for injections) available and used specifically for each age group?	Yes
	No
Are the buildings/rooms cleaned and disinfected after each production cycle?	Yes
	No
Are the corridors cleaned and disinfected after moving animals?	Not always
	Always
Are footbaths present at the entrance of the farm, and are they used?	Yes
	No
Is the liquid in the footbath changed immediately when it becomes visibly contaminated?	Yes
	No
	No footbath

List of exploratory variables included in the automatic model selection analysis for the bio-compartmentalization compartment. Bio-compartmentalizing measures are measures preventing the inter-animal spread and dissemination of a pathogen on a given farm.
